# Severe Multivessel Coronary Vasospasm From Hypovolemia: A Case Report

**DOI:** 10.7759/cureus.63316

**Published:** 2024-06-27

**Authors:** Fawaz Mohammed, Sajjad Haider, Mohammed Kazimuddin, Mohammad Abdul-Waheed, Muhammad Akbar

**Affiliations:** 1 Internal Medicine, University of Kentucky College of Medicine, Bowling Green, USA; 2 Cardiology, University of Kentucky College of Medicine, Bowling Green, USA; 3 Internal Medicine, Medical Center at Bowling Green, Bowling Green, USA

**Keywords:** coronary artery angiography, mimicking st elevation, severe hypovolemia, coronary artery vasospasm, st-segment–elevation

## Abstract

Cases of coronary vasospasm leading to ST-elevation myocardial infarction (STEMI) have been described. However, hypovolemia as an etiology of coronary vasospasm has been rarely reported. We report the case of a 57-year-old male who presented to the hospital with syncope, with electrocardiogram (EKG) findings suggestive of ST elevation in the inferior leads. The catheterization lab was activated, and coronary angiography was performed, which showed no evidence of thrombotic occlusion with diffuse disease in the right coronary artery and left anterior descending artery that resolved with aggressive fluid resuscitation.

## Introduction

Myocardial infarction with ST elevations occurs because of atherosclerotic plaque rupture. Coronary vasospasm is another life-threatening etiology of ST-elevation myocardial infarction (STEMI) that can have detrimental complications. However, dehydration as a cause of ST elevation from coronary vasospasm has rarely been reported. We report the case of a 57-year-old male who presented to the hospital with a syncopal episode with EKG findings consistent with inferior wall ST-elevation myocardial infarction. Coronary angiography was performed, which showed the right coronary artery (RCA) and left anterior descending artery (LAD) vasospasm that resolved with fluid resuscitation.

## Case presentation

**Figure 1:**
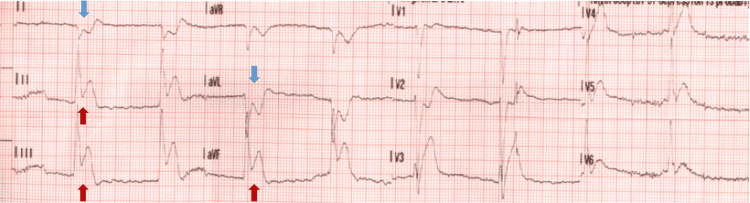
EKG findings EKG showing ST elevations in leads II, III, and aVF (red arrows) with reciprocal changes in lead I and aVL (blue arrows).

**Figure 2:**
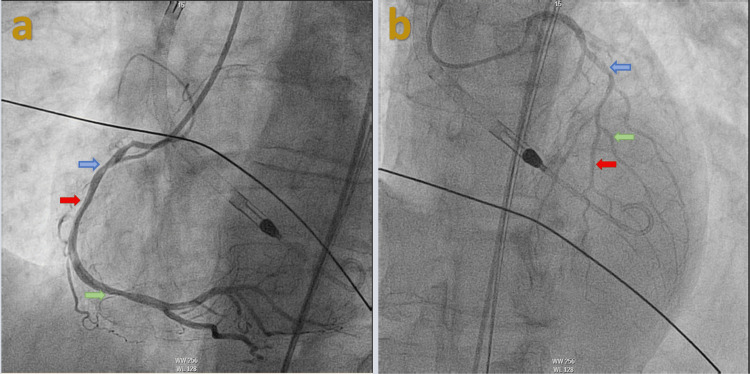
Coronary angiography findings before fluid resuscitation Coronary angiography with (a) left anterior oblique (LAO) cranial view showing proximal RCA with 80% stenosis (blue arrow), mid-RCA with 60% stenosis (red arrow), distal RCA stenosis with 60% stenosis (green arrow), (b) anteroposterior (AP) cranial view showing 80% stenosis in the mid-LAD (blue arrow), tapered distal LAD (red arrow), and diagonal (green arrow), which are both small caliber vessels.

**Figure 3:**
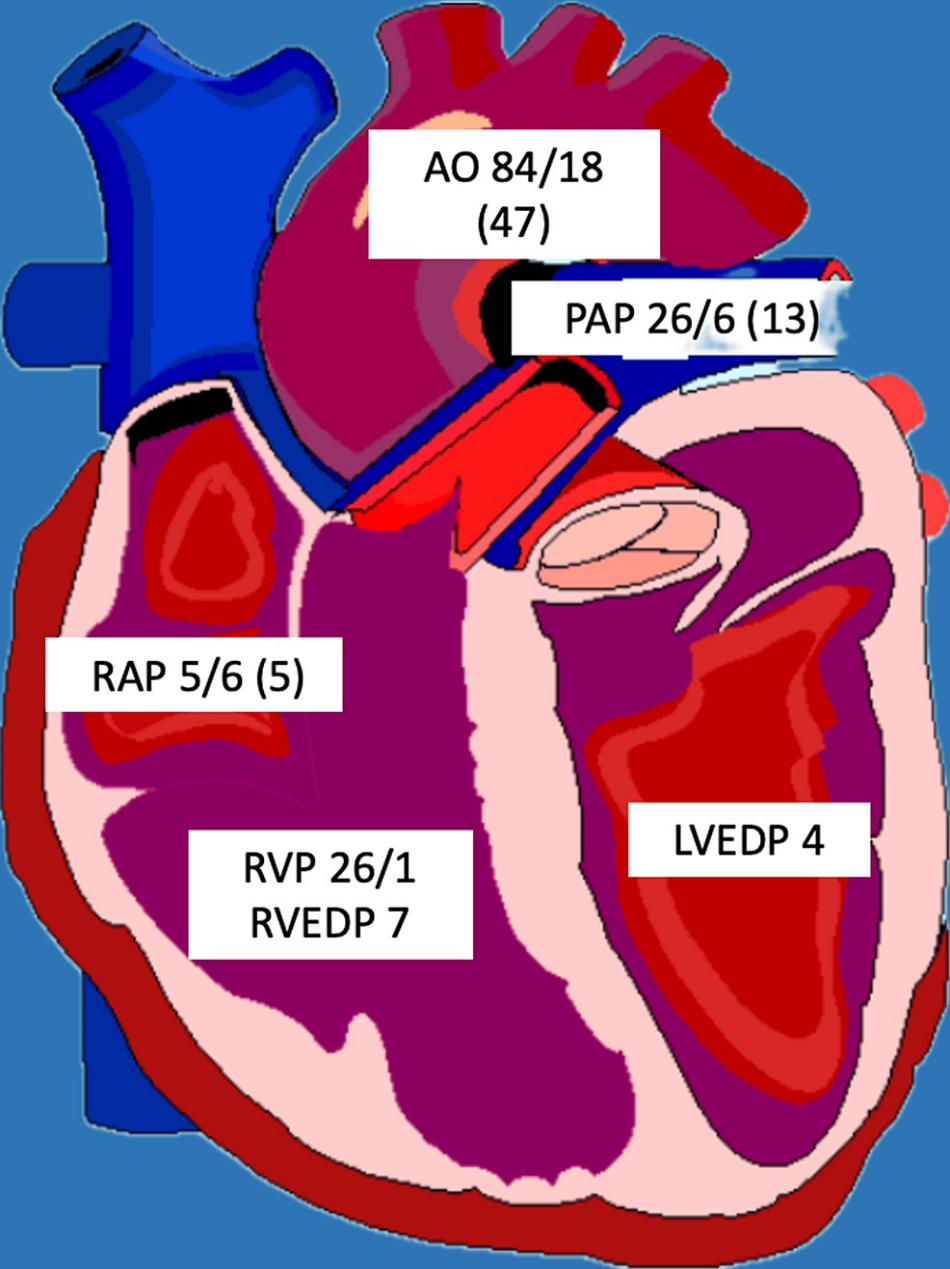
Right heart catheterization findings All units are expressed in millimeters of mercury (mmHg). Aortic pressure (AO), right atrial pressure (RAP), right ventricular pressure (RVP), right ventricular end-diastolic pressure (RVEDP), pulmonary artery pressure (PAP), and left ventricular end-diastolic pressure (LVEDP).

**Figure 4:**
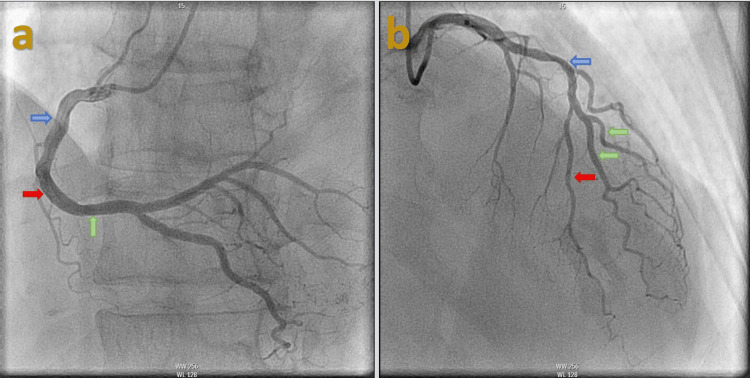
Coronary angiography findings after aggressive fluid resuscitation Repeat coronary angiography showing (a) proximal (blue arrow), mid (red arrow), and distal (green) RCA with a resolution of stenosis following fluid resuscitation, (b) mid-LAD artery with about 30% stenosis (blue arrow), distal LAD artery (red arrow), and diagonal branches (green arrows) larger in caliber compared to the first coronary angiogram.

A 57-year-old male was brought to the hospital via EMS after he suffered a syncopal event while driving. His past medical history was significant for hypertension. Social history was significant for active tobacco use with smoking about half a pack a day for the past 43 years. He denied a history of illicit drug use, and his urine drug screen was negative. He was noted to be diaphoretic on presentation; however, he denied any chest pain or palpitations before the syncopal event. He did report shortness of breath that was chronic for him that he attributed to his tobacco use history. Of note, he reported watery stools for the past two weeks. He did report lower abdominal pain diffusely that was sharp. He denied any nausea or vomiting. On presentation, he was noted to be hypotensive with a blood pressure of 72/43 mmHg and a heart rate of 103 per minute. He was noted to be slightly confused and agitated on physical examination. No murmurs or abnormal heart sounds were noted. EKG revealed marked ST elevations in the inferior leads (Figure 1). The cardiac catheterization laboratory was activated, and the patient was taken for emergent heart catheterization. Coronary angiography revealed a dominant RCA with multiple tandem lesions in the proximal, middle (mid), and distal RCA (Figure 2a). The mid-LAD showed stenosis along with tapering of the distal LAD and diagonal branches (Figure 2b). Right heart catheterization (RHC) revealed pulmonary capillary wedge pressure of 10/15 mmHg, mean of 9 mmHg, pulmonary artery pressure of 26/6 mmHg; mean of 13 mmHg, right ventricular pressure of 26/1 mmHg, right ventricular end-diastolic filling pressure of 7 mmHg, and right atrial pressure of 5/6 mmHg; mean of 5 mmHg and left ventricular end-diastolic pressure was 4 mmHg (Figure 3). Cardiac output via the Fick method was 5.05 L/min, the cardiac index was 3.1 L/min/m^2^ with pulmonary vascular resistance of 63 dynes x sec/cm5, and systemic vascular resistance was 206 dynes x sec/cm^5^. RHC findings were consistent with low-filling pressures, and normal cardiac output, with no evidence of pulmonary hypertension. The patient was then transferred to a cardiac care unit where he was managed with aggressive intravenous fluid resuscitation and pressor support with improvement in hemodynamics. With multivessel disease noted on initial coronary angiography repeat coronary angiography was performed to evaluate the RCA and LAD with fractional flow reserve (FFR). The RCA and LAD showed no significant stenosis compared to the initial angiogram which was likely because of coronary vasospasm in the setting of hypovolemic shock (Figures 4a-4b). The patient was transferred back to the cardiac care unit with no episodes of chest pain, syncope, or arrhythmias. His home hydrochlorothiazide was discontinued, and he was discharged with aspirin 81 milligrams (mg) daily, atorvastatin 40 mg daily, and metoprolol 12.5 mg twice daily in stable condition.

## Discussion

Spasm of the coronary artery is a frequently described phenomenon as a cause of angina [1]. Damage to the myocardium can occur when the spasm is severe enough or occurs for a protracted period. Although the mechanism of spasm is not completely understood, hypersensitivity of the coronary arteries to mediators of vasoconstriction is one proposed mechanism. Etiologies of the vasospasm are related to the abnormalities of the endothelium, elevated levels of products of inflammation, and deficient magnesium levels [2]. The literature has also suggested hyperthyroidism as a cause of coronary vasospasm [3].

Review of literature has suggested that negative fluid balance (dehydration) is a marker for the development of life-threatening coronary artery disease [4]. The blood viscosity of whole blood is influenced by hemoconcentration, which is a marker of dehydration. Increased blood viscosity leads to shear stress on the endothelial lining thereby causing the progression of atherosclerosis [5]. Another proposed mechanism describing the mechanism of dehydration causing coronary artery spasm is the underfilling of coronary arteries in hypovolemic states [6].

Obstructive coronary artery disease should be considered in patients with coronary vasospasm [7]. The prognosis of individuals with infarction from coronary vasospasm is generally favorable [8].

Medical management comprises of calcium channel blockers and nitrate therapy. In cases of multivessel spasms, calcium channel blockers are recommended to be continued to decrease the risk of arrhythmias [2].

In cases of severe vasospasm, mechanical device support may be required as cases may be complicated with life-threatening arrhythmias [9]. Sudden cardiac death can occur in approximately 10% of individuals. The literature has also suggested the use of implantable cardioverter-defibrillator (ICD) in patients with cardiac arrest from this etiology, although improved mortality has not been seen with ICD use [10]. Of note, in patients with cardiac arrest of unknown etiology, coronary vasospasm as a cause should always be considered [11]. Multivessel coronary vasospasm has been shown to have a poorer prognosis as compared to the involvement of a single vessel [12].

Other classes of medications that are beneficial are endothelin-receptor antagonists [13].

Percutaneous coronary artery intervention may be of utility in individuals with significant coronary artery disease [2]. Given there was no presence of significant coronary artery disease in our patient, intervention with intracoronary stenting was not pursued.

## Conclusions

Coronary vasospasm as an etiology of chest pain should be considered in patients presenting with ST-segment elevation with no risk factors for atherosclerotic cardiovascular disease. Severe dehydration as an etiology of coronary vasospasm is a rarely described phenomenon. Aggressive resuscitation with maintenance of fluid balance is of utmost importance in patients with this rare presentation.
